# Efficacy of Xianling Gubao capsule in treating sarco-osteopenia: Protocol for a systematic review and meta-analysis

**DOI:** 10.1097/MD.0000000000015672

**Published:** 2019-05-17

**Authors:** Qunqun Chen, Junxiang Zeng, Yi Chen, Yalan Yang, Tian Zhang, Tao Luo, Hongxing Huang

**Affiliations:** a The Third Affiliated Hospital of Guangzhou University of Chinese Medicine; b Guangzhou University of Chinese Medicine, Guangzhou; c ZhaoQing City's Traditional Chinese Medical Hospital, ZhaoQing, China.

**Keywords:** protocol, sarco-osteopenia, systematic review, Xianling Gubao capsule

## Abstract

**Background:**

Sarco-osteopenia (SOP) is a new type of geriatric syndrome, resulting from the combination of sarcopenia (SP) and osteoporosis (OP). Xianling Gubao capsule (XLGBC), made from several traditional Chinese medicine, is reported to have a therapeutic effect on diseases of bones and joints. This protocol will be designed to assess the efficacy of XLGBC in the treatment of SOP.

**Methods:**

Relevant randomized controlled trial literatures evaluating the effect of XLGBC on patients with SOP will be obtained by searching the following 7 electronic databases: Cochrane Library, PubMed, Embase, Chinese National Knowledge Infrastructure (CNKI), Chinese Scientific Journal Database (VIP), Chinese Biomedical and Medical Database (CBM), and Wanfang Database, from inception to March 2019. The primary outcomes will be bone mineral density, skeletal muscle mass index, handgrip strength, and gait speed. Stata V.13.0 software will be used for data synthesis and analysis, sensitivity analysis, subgroup analysis, and risk of bias assessment. Reporting bias will be evaluated utilizing a funnel, with Egger tests assessing funnel plot symmetries. Quality of evidence will be evaluated according to guidance of the Recommendations Assessment, Development, and Evaluation guideline.

**Result:**

This study will provide a rational synthesis of current evidences for XLGBC on SOP.

**Conclusion:**

The conclusion of this study will provide evidence to judge the effectiveness and safety of XLGBC on SOP.

**Ethics and dissemination:**

This systematic review will be contributed to peer-reviewed publications, aiming to provide evidence about efficacy of XLGBC on SOP.

**Trial registration number:**

CRD42019128223.

## 1 Introduction

### 1.1 Description of the condition

Because of the aging group, this fraction epidemic is predicted to exponentially escalate by 2050.^[1]^ Sarcopenia (SP) and osteoporosis (OP) are 2 age-related chronic diseases. SP is diagnosed as the decline in skeletal muscle mass and function, which has serious influence on patient's activities and diet. OP is defined as a systemic bone disease characterized by age-related fracture risk. Loss of bone and muscle strength combine into a single diagnosis of “sarco-osteopenia” (SOP).^[2]^ SOP is a major disease for elderly people, which is considered as a higher risk for falls and fractures.^[2–4]^ Among elderly people, SOP is associated with significant morbidity and mortality as well as a decline in the quality of life. After 50 years, muscle mass decreases by 0.5% to 2% annually and muscle strength by 1.5% to 3%.^[5]^ This decrease is greater in the sedentary population and in males, in whom a prevalence of double resistance is found. Also, for women, bone mass begins to decrease by 0.5% from 30 years with a rapid point decrease in postmenopausal women, this decrease being stable in men.^[6,7]^ These changes together with multiple contributing factors, such as sedentary lifestyle, malnutrition, chronic diseases, and some pharmacological treatments, finally producing OP and SP, even SOP.

### 1.2 Description of the intervention

As for treatments for SOP, new pharmacological treatments are developed for the management of low body mass index (BMI) and SP.^[8]^ Myostatin (inhibitor of muscle development) is one of the therapeutic targets that are currently studied.^[9]^ A study showed that administration of ACVR2B-Fc (myostatin receptor recombinant protein) resulted in an increase in lean body mass and markers of bone formation in postmenopausal women.^[10]^ The antibody LY2495655 has been associated with a moderate increase in muscle mass and strength.^[11]^ Other pharmacological therapies such as testosterone, inhibitors of angiotensin-converting enzyme, ghrelin, growth hormone, and insulin-like factor 1 have also been evaluated to treat both OP and SP, but no clear evidence of their benefits has been found so far.^[12]^ Chinese medicine has good clinical efficacy in the treatment of OP. And Xianling gubao is the well-known Chinese patent medicine. The recipe is based on the modern prescriptions of the Miao people. A new type of national medicine was developed. The prescription was collected, sorted, and screened by a famous orthopedics expert, Professor Shi Guangda, and eventually consisted of 9 Chinese herbs, such as epimedium, psoralen, psoralen, Radix Rehmanniae, Salvia miltiorrhiza, and Zhimu, which are mainly used in the treatment of OP, fracture, osteoarthritis, aseptic necrosis, etc. There are a lot of literatures showing that Xianling Gubao capsule (XLGBC) has a good effect on the treatment of bone diseases.^[13–15]^ Thus, the purpose of this review is to summarize clinical researches on XLGBC for SOP and findings of this review will be reliable within evidence of clinical studies.

## 2 Methods and analysis

### 2.1 Study registration

**Figure 1:**
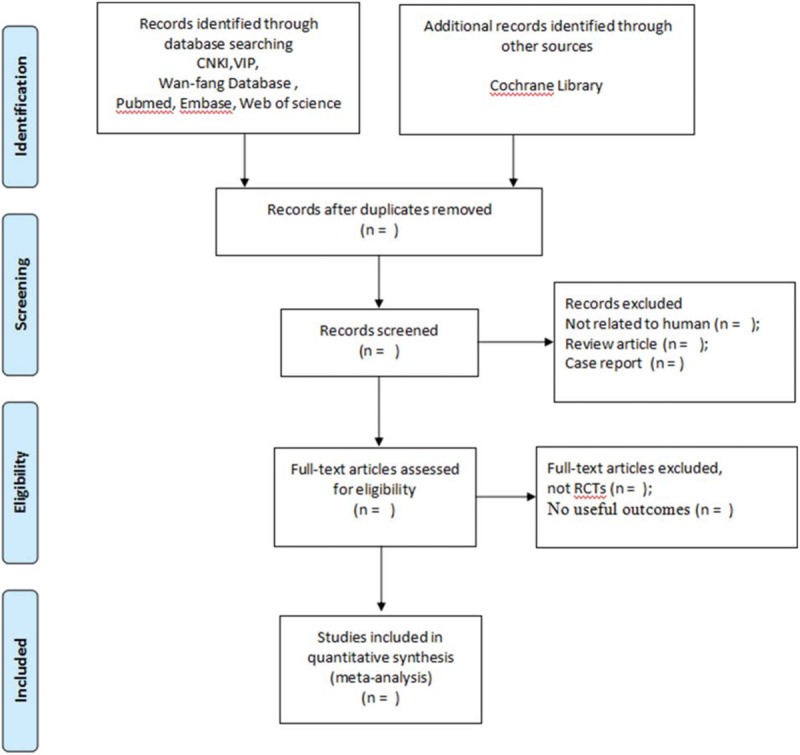
PRISMA 2009 flow diagram.

The protocol has been registered on the International Prospective Register of Systematic Reviews (PROSPERO) (registration number, CRD42019128223) basing on the Preferred Reporting Items for Systematic Reviews and Meta-Analyses Protocols (PRISMA-P) statement guidelines (Fig. 1).

### 2.2 Eligibility criteria

#### 2.2.1 Types of studies

Regardless of the blind method and language, randomized controlled trials that evaluate the effect of XLGBC on patients with SOP will be included. The following types of studies will be excluded: nonclinical researches, including animal experiments, reviews, conference papers, and so on; duplicate publications; literatures lacking data that are needed for our study; studies that have no diagnostic criteria or efficacy criteria; and the baseline data are significantly inconsistent.

#### 2.2.2 Types of participants

This study will include patients who simultaneously meet the diagnosis of OP and SP.

#### 2.2.3 Types of interventions

The control group will comprise patients receiving no treatment, placebo, or routine treatment. The intervention group will include XLGBC based on routine regimens as intervention measures.

#### 2.2.4 Types of outcomes measures

The primary outcomes will include BMD, SMI, handgrip strength, and gait speed.

The secondary outcomes will include short physical performance battery score (SPPBS), balance ability, pain, and adverse events.

### 2.3 Search strategy

#### 2.3.1 Electronic searches

Seven databases including Cochrane Library, PubMed, Embase, Chinese National Knowledge Infrastructure (CNKI), Chinese Scientific Journal Database (VIP), Chinese Biomedical and Medical Database (CBM), and Wanfang Database will be searched with a time span from their inception to March 2018. The search strategy in PubMed database is as follows: “Xianling Gubao Capsule” AND “Sarco-Osteopenia” or (“sarcopenia” and “osteoporosis”). Chinese translation of the above retrieval strategy will be used for Chinese database search.

#### 2.3.2 Other resources

In order to obtain the potential nonelectronic literatures, relevant magazines and medical journals will also be filtered for further search.

#### 2.3.3 Study selection and data extraction

With the help of Endnote V.X7 (Columbia CP Ltd), 2 reviewers will carry out the first round of selection by preliminarily screening the title and abstract of the obtained articles. Duplicate publications, nonclinical researches, and irrelevant articles will be excluded. Another round of selection will be conducted through reading the full text of the documents to decide the final inclusions. Any disagreement will be settled by discussion the judgement of a third reviewer. The study selection procedure will be showed by a Preferred Reporting Items for Systematic Reviews and Meta-Analyses (PRISMA) flow chart (Fig. 1). Microsoft Excel will be utilized for data extraction. Data including the following items, general information, trial characteristics, participants, interventions, and outcomes, will be collected in 5 spreadsheets, respectively.

#### 2.3.4 Dealing with the missing data

If any article lacks the necessary data, we will contact authors of the article by the contact information we can get. If we fail in this way, the potential impact that the missing data would probably bring about will be analyzed in the discussion part.

#### 2.3.5 Risk of bias in included studies

According to suggestions from Cochrane Handbook for Systematic Reviews of Interventions, risk of bias of each included trial will be assessed in the following aspects: random sequence generation, allocation concealment, implementation of the blind method, incomplete data, selective report, and other bias. Risk of bias will be evaluated as low, high, or unclear (unclear or unknown risk of bias).

### 2.4 Statistical analysis and data synthesis

Meta-analysis of the included studies will be performed by statistical softwares, respectively (Stata 14.0 software (Canada, Statagroup) and Stata 12.0 software (Canada, Statagroup)). Mean difference (MD) or standard mean difference (SMD) will be used for continuous variables, and rate ratio (RR) will be chosen for dichotomous variables. Besides, 95% confidence intervals (95% CIs) for both variables will also be measured. As for data synthesis, the heterogeneity of each included studies will be assessed by *I*^2^ test statistics firstly. The evaluation criteria are as follow: *I*^*2*^ < 25% means no significant heterogeneity, *I*^*2*^ = 25% to 50% indicates moderate heterogeneity, and *I*^*2*^ > 50% represents strong heterogeneity. Then, basing on the *I*^*2*^ value, the random-effects model (*I*^*2*^ ≥ 50%, significant heterogeneity) or the fixed-effect model (*I*^*2*^ < 50%, low heterogeneity) will be chosen.

### 2.5 Subgroup analysis and sensitivity analysis

Once heterogeneity appears significant (*I*^*2*^ ≥ 50%) and the trials included are adequate, we will perform subgroup analysis and sensitivity analysi**s** to seek the probable source of the heterogeneity, in consideration of different study characteristics, such as participants characteristics, sample size, interventions, controls, outcome measures, and so on.

### 2.6 Assessment of reporting bias

When the number of the included studies exceeds 10, reporting bias will be assessed by funnel plots drawn through Egger regression test. Symmetry of funnel plots indicates no reporting bias. On the contrary, if the points of the funnel plot appear to be dispersed and asymmetrical, reporting bias is considered to be existent and the reliability is low.

### 2.7 Quality of evidence

With the guidance of the Recommendations Assessment, Development, and Evaluation guideline, the quality of evidence will be evaluated by 2 reviewers. We will take into account limitations of the study, inconsistencies, indirect evidence, imprecision, and publication bias. Levels of evidence quality will be rated as high, moderate, low, or very low.

### 2.8 Ethics and dissemination

Ethical approval will not be needed, as the data extracted for our study are derived from published literature and will not cause invasion of participant privacy. We aim to publish this systematic review, evaluating the effects of XLGBC on SOP.

## 3 Discussion

Although currently in SOP drug research has made great progress, a common target for “muscle-osteoporosis” therapeutic drugs is still less, and the efficacy is not exact.^[16]^ The current treatments are single treatments for one of these conditions.^[17]^ May be beneficial to the muscles at the same time, and therefore extended to treat the muscles less serious. It mainly includes anabolic hormones, vitamin D, receptor agonists, and growth hormones.^[18]^

To the best of our knowledge, it will be the first systematic review and meta-analysis on XLGBC in the treatment of SOP. First, the results of this review will provide objective statistics for further researches on SOP. Second, the results will offer reliable references for clinicians and patients in the treatment of SOP with XLGBC. Third, the results may introduce an alternative therapy of SOP to policy makers to decrease the burden of public health.

## Author contributions

Hongxing Huang conceived the study idea. Qunqun Chen was responsible for the design of this systematic review. Junxiang Zeng and Yi Chen contributed to the data analysis plan. Yalan Yang and Tian Zhang drafted the manuscript and Tao Luo edited it. All authors provided feedback and approved the final manuscript.

**Data curation:** Qunqun Chen.

**Formal analysis:** Qunqun Chen.

**Investigation:** Junxiang Zeng.

**Methodology:** Yi Chen.

**Software:** Yalan Yang, Tian Zhang.

**Writing – original draft:** Tao Luo.

**Writing – review & editing:** Hongxing Huang.

## References

[1] Amin S Achenbach SJ Atkinson EJ Trends in fracture incidence: a population-based study over 20 years. J Bone Miner Res 2014;29:581–9. 23959594 10.1002/jbmr.2072 PMC3929546

[2] Binkley N Buehring B Beyond F RAX: it's time to consider “Sarco-Osteopenia”. J Clin Densitom 2009;12:413–6. 19733110 10.1016/j.jocd.2009.06.004

[3] Demontiero O Boersma D Suriyaarachchi P Clinical outcomes of impaired muscle and bone interactions. Clinic Rev Bone Miner Metab 2014;12:86–92.

[4] Coin A Perissinotto E Enzi G Predictors of low bone mineral density in the elderly: the role of dietary intake, nutritional status and sarcopenia. Eur J Clin Nutr 2008;62:802–9. 17637603 10.1038/sj.ejcn.1602779

[5] Alonsobouzon C Duque G [Senile osteoporosis: an update]. Rev Esp Geriatr Gerontol 2011;46:223–9. 21601957 10.1016/j.regg.2011.02.010

[6] Rizzoli R Branco J Brandi ML Management of osteoporosis of the oldest old. Osteoporos Int 2014;25:2507–29. 25023900 10.1007/s00198-014-2755-9

[7] Sakuma K Yamaguchi A Recent advances in pharmacological, hormonal, and nutritional intervention for sarcopenia. Pflugers Arch 2017;470:1–2. 29043432 10.1007/s00424-017-2077-9

[8] Dhillon RJ Hasni S Pathogenesis and management of sarcopenia. Clin Geriatr Med 2017;33:17. 27886695 10.1016/j.cger.2016.08.002 PMC5127276

[9] Attie KM Borgstein NG Scd YY A single ascending-dose study of muscle regulator ace-031 in healthy volunteers. Muscle Nerve 2013;47:416–23. 23169607 10.1002/mus.23539

[10] Becker C Lord SR Studenski SA Myostatin antibody (LY2495655) in older weak fallers: a proof-of-concept, randomised, phase 2 trial. Lancet Diabetes Endocrinol 2015;3:948–57. 26516121 10.1016/S2213-8587(15)00298-3

[11] Snyder PJ Bhasin S Cunningham GR Effects of testosterone treatment in older men. N Engl J Med 2016;374:611–24. 26886521 10.1056/NEJMoa1506119 PMC5209754

[12] Berenbaum F Griffin TM Liu-Bryan R Review: metabolic regulation of inflammation in osteoarthritis. Arthritis Rheumatol 2017;69:9–21. 27564539 10.1002/art.39842 PMC5341385

[13] Qi X Ren J Effects of two kinds of Xianling Gubao extract on liver mitochondria. Chin J Pharmacol 2016;14:201.

[14] Duolin H A review of the research on the treatment of osteoporosis with Xianling Gubao capsule. J Trad Chin Med 2013;28:285.

[15] Sun L Tang H Meta-analysis of the efficacy and safety of Xianling Gubao capsule in the treatment of osteoporotic pain. Chin Med J 2016;13:422.

[16] Morley JE Pharmacologic options for the treatment of sarcopenia. Calcif Tissue Int 2016;98:319–33. 26100650 10.1007/s00223-015-0022-5

[17] Vanderschueren D Laurent MR Claessens F Sex steroid actions in male bone. Endocr Rev 2014;35:906–60. 25202834 10.1210/er.2014-1024 PMC4234776

[18] Rose KA Murphy CA Diamond SL Growth hormone replacement therapy prevents sarcopenia by a dual mechanism: improvement of protein balance and of antioxidant defenses. J Gerontol 2014;69:1186–98. 10.1093/gerona/glt187 24300031

